# Repurposing Screens of FDA-Approved Drugs Identify 29 Inhibitors of SARS-CoV-2

**DOI:** 10.4014/jmb.2009.09009

**Published:** 2020-11-10

**Authors:** Keun Bon Ku, Hye Jin Shin, Hae Soo Kim, Bum-Tae Kim, Seong-Jun Kim,, Chonsaeng Kim

**Affiliations:** Center for Convergent Research of Emerging Virus Infection, Korea Research Institute of Chemical Technology, Daejeon 34114, Republic of Korea

**Keywords:** SARS-CoV-2, COVID-19, FDA-approved drugs, repurposing screens

## Abstract

COVID-19, caused by the novel coronavirus SARS-CoV-2, has spread globally and caused serious social and economic problems. The WHO has declared this outbreak a pandemic. Currently, there are no approved vaccines or antiviral drugs that prevent SARS-CoV-2 infection. Drugs already approved for clinical use would be ideal candidates for rapid development as COVID-19 treatments. In this work, we screened 1,473 FDA-approved drugs to identify inhibitors of SARS-CoV-2 infection using cell-based assays. The antiviral activity of each compound was measured based on the immunofluorescent staining of infected cells using anti-dsRNA antibody. Twenty-nine drugs among those tested showed antiviral activity against SARS-CoV-2. We report this new list of inhibitors to quickly provide basic information for consideration in developing potential therapies.

## Introduction

The recent spread of severe acute respiratory syndrome coronavirus 2 (SARS-CoV-2) has caused an outbreak of the emerging disease COVID-19 (coronavirus disease 2019) [1]. The World Health Organization (WHO) declared this outbreak a pandemic in March 2020 [2]. This pandemic is causing serious social and economic problems [3]. The symptoms of COVID-19 patients vary widely, from mild illness to severe diseases including dyspnea, pneumonia, and ultimately death [4-6]. SARS-CoV-2 belongs to the β coronaviruses, two of which are potentially fatal, Middle East respiratory syndrome coronavirus (MERS-CoV) and SARS-CoV [7-9]. No effective vaccines or antiviral drugs are currently available, although they are urgently needed. Conventional drug development is a costly and time-consuming process that probably will not be able to achieve timely development of COVID-19 drugs. Drug repurposing is an alternative approach, able to rapidly identify new treatments for COVID-19 [10]. Drugs already approved for clinical use would be ready for use sooner, owing to their established safety profile and pharmacokinetics. Anti-HIV drugs and remdesivir, a nucleotide analogue, have been tested for the treatment of COVID-19 [11, 12].

In this study we screened 1,473 food and drug administration (FDA)-approved drugs using a cell-based assay of SARS-CoV-2 infection. This screening identified 29 drugs with anti-viral effect against SARS-CoV-2. This study offers a new list of drugs for consideration as potential therapies.

## Materials and Methods

### Viruses and Cells

SARS-CoV-2 (BetaCoV/Korea/KCDC03/2020) was provided by the Korea Centers for Disease Control and Prevention (NCCP43326). Vero cells (CCL-81) were purchased from the American Type Culture Collection (ATCC) and maintained in Dulbecco’s modified Eagle medium (DMEM) supplemented with 10% fetal bovine serum (both HyClone, USA). SARS-CoV-2 was expanded in the Vero cells and titers were determined by plaque assay. All experiments were performed in a Biosafety Level 3 (BSL3) facility.

### Chemical Library and Antibodies

The FDA-approved drug library of 1,473 drugs and remdesivir were provided by the Korea Chemical Bank at the Korea Research Institute of Chemical Technology (KRICT). The J2 anti-double-stranded RNA (dsRNA) antibody was purchased from English and Scientific Consulting Kft. (Szirák, Hungary, catalog no.10010200). Alexa Fluor 488-conjugated goat anti-mouse antibody was obtained from Life Technologies (USA, catalog no. A11001).

### Antiviral Screen

One day before infection, Vero cells were seeded in 96-well plates at a density of 1 × 10^4^ cells per well. Each compound was added at 20 μM final concentration. Cells were then infected with SARS-CoV-2 at an MOI of 0.5. Three days after infection, the cytopathic effects (CPE) of the virus were determined by microscopic examination. Drugs showing strong inhibition of viral CPE were selected and their antiviral efficacy was further investigated (see next section).

After optimization of CPE-inhibition assay for screening, Vero cells were seeded as the first screening. Each compound was diluted in DMEM including 1 g/mL glucose (low glucose condition) in absence of fetal bovine serum and added to cells at 20 μM final concentration. Cells were infected with SARS-CoV-2 at an MOI of 0.5 using the same media used for compound dilution. Three days after infection, cell viability was measured by colorimetric assay using MTS solution (Promega, USA, catalog no. G3582). Assay results from mock infected cells were treated as the 100% survival standard; those for virus-infected cells in the absence of drugs were considered as the 0% survival standard. The antiviral activity for each tested drug was calculated as the percentage cell survival by comparison with these standards.

### Antiviral Activity and Cell Toxicity Determination

The evaluation of antiviral activity and cell toxicity was conducted on drugs that showed anti-SARS-CoV-2 potential in the antiviral screen described above. We seeded 2 × 10^4^ Vero cells per well in 96-well plates one day before infection. Serially diluted drugs (100 μM to 0.16 μM) were added to the cells 1 hour prior to infection. The cells were infected with SARS-CoV-2 at an MOI of 2. At 24 h post-infection, cells were fixed with 4%paraformaldehyde and permeabilized with 0.1% Triton X-100 in phosphate-buffered saline. The cells were then stained with anti-dsRNA antibody and Alexa-Fluor 488-conjugated secondary antibody. Their nuclei were counterstained with Hoechst 33342 (Thermo Fisher Scientific, USA, catalog no. H3570). Image acquisition and analysis were performed as previously described [13]. Viral infection was quantified by dividing the number of cells stained with anti-dsRNA antibody by the total number of cells (obtained by counting the nuclei). Infection rate standards were obtained from mock infected cells (0%) and drug-free SARS-CoV-2 infected cells (100%). Infection rates in drug-treated cells were calculated based on these standards. Drug antiviral activity was determined from dose-response curves; half maximal inhibitory concentration (IC_50_) values were calculated using Prism v8 software (GraphPad Software). To determine the cell toxicity (CC_50_), similar experiments were performed without addition of the virus; cell viability was measured using MTS solution. CC_50_ values were calculated using Prism. The selectivity index (SI) was calculated as CC_50_/IC_50_.

## Results and Discussion

**Fig. 1:**
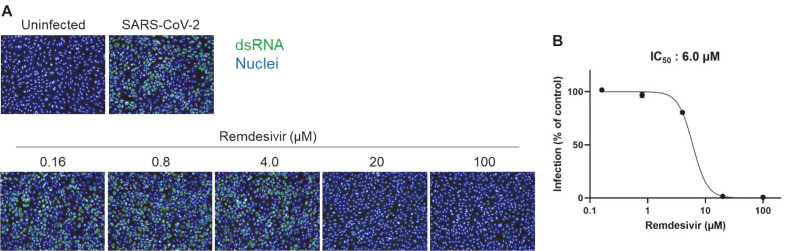
Antiviral assay based on immunofluorescent staining using anti-dsRNA antibody showed that remdesivir potently inhibits SARS-CoV-2 infection. (**A**) Vero cells were treated with increasing concentrations of remdesivir and infected with SARS-CoV-2 (2 MOI). Twenty-four hours post-infection, infected cells were stained with anti-dsRNA antibody and visualized with Alexa Fluor 488-conjugated secondary antibody (green). Nuclei were stained with Hoechst 33342 (blue). (**B**) Virus infection was calculated by counting the stained cells and antiviral activity (IC_50_) was determined from the dose-response curve.

**Fig. 2:**
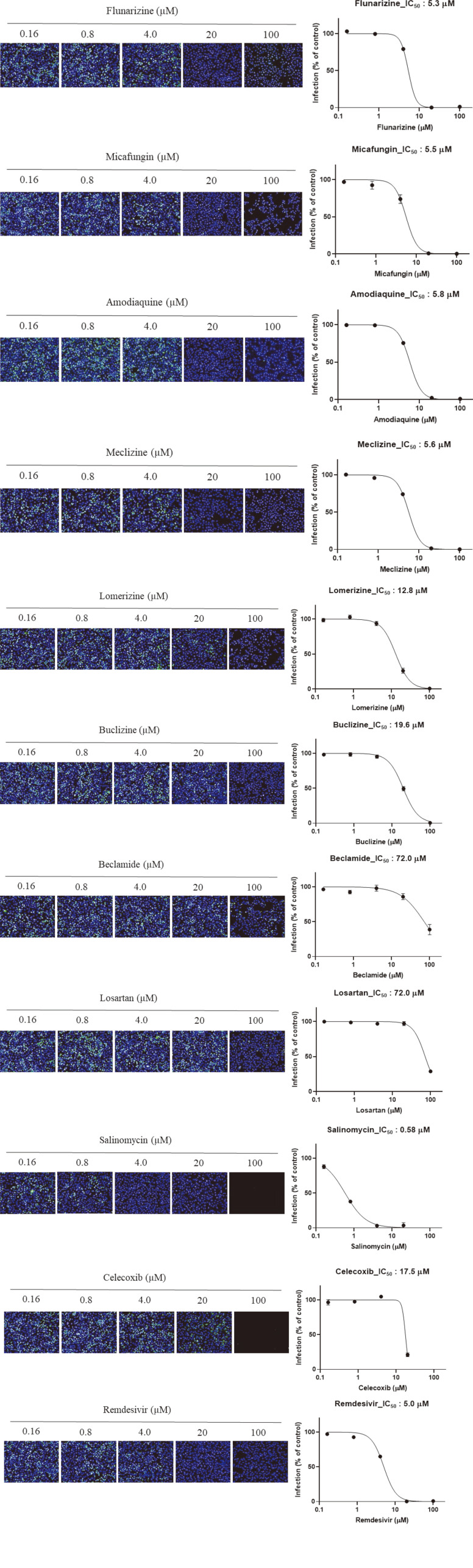
Dose-response curve of 10 compounds selected by the primary screening with remdesivir as a positive control. The microscope images show cell nuclei (blue) and dsRNA (green) at each drug concentration.

As SARS-CoV-2 did not efficiently induce cell death, the antiviral assay based on cell survival was unable to produce consistent results. Further optimization to efficiently induce cell death was required in order to measure antiviral activity based on cell survival. However, it was possible to measure the antiviral effect by observing the cells under a microscope. Observations were calibrated using the clear CPE in untreated cells infected with SARS-CoV-2 and complete absence of CPE in the presence of the reference compound remdesivir, which has been reported as an inhibitor of SARS-CoV-2 [14]. During the optimization of the antiviral assay, a primary antiviral screening of 1,473 FDA-approved drugs was performed at a final concentration of 20 μM based on the microscopic observations. Ten compounds showed strong inhibition of CPE, similar to that seen with remdesivir. To confirm the antiviral activity of these ten compounds, we used an antiviral assay based on immunofluorescent staining using anti-dsRNA antibody calibrated using a reference compound, remdesivir. This antibody has been used for detection of dsRNA in cells infected with diverse positive-strand RNA viruses including SARS-CoV [15]. As shown in Fig. 1, SARS-CoV-2-infected cells showed a strong fluorescent signal, which gradually decreased in a dose-dependent manner under treatment with remdesivir. The IC_50_ was 6 μM. Next, the ten compounds selected by the primary screen were tested over a broader concentration range. All ten candidates showed antiviral activity against SARS-CoV-2 (Table 1 and Fig. 2). The IC_50_ values are summarized in Table 1, which also shows the cell toxicity (CC_50_) and selectivity index (SI) values for these ten compounds.

The compounds were flunarizine, micafungin, amodiaquine, meclizine, lomerizine, buclizine, beclamide, losartan, salinomycin, and celecoxib. Flunarizine is a calcium channel blocker and a prophylactic drug for migraine [16]. Lomerizine is a voltage-dependent calcium channel antagonist and is also used as a prophylactic drug for migraines [17]. Previous reports, based on screenings of FDA-approved drugs, have identified flunarizine and lomerizine as inhibitors of Japanese encephalitis virus [18]. Flunarizine is able to inhibit hepatitis C virus cell entry and membrane fusion [19] and lomerizine has shown antiviral activity against Ebola virus [20]. Interestingly, it was recently reported that calcium ions directly interact with the Ebola virus fusion peptide to enhance viral infection; calcium-interfering drugs could therefore be used as therapeutics for this virus [21]. Our results and previous reports suggest that calcium ions could also be important for SARS-CoV-2 entry and membrane fusion. Meclizine and buclizine have been widely used to treat motion sickness and are piperazine-derived histamine H1 antagonists [22-24]. There are no reports on the antiviral effects of these two drugs. Flunarizine, lomerizine, meclizine, and buclizine are similar in structure, with a piperazine moiety. Further analysis of the structure-activity relationships of these four inhibitors would be helpful in understanding the molecular structure responsible for their antiviral activity against SARS-CoV-2.

**Fig. 3:**
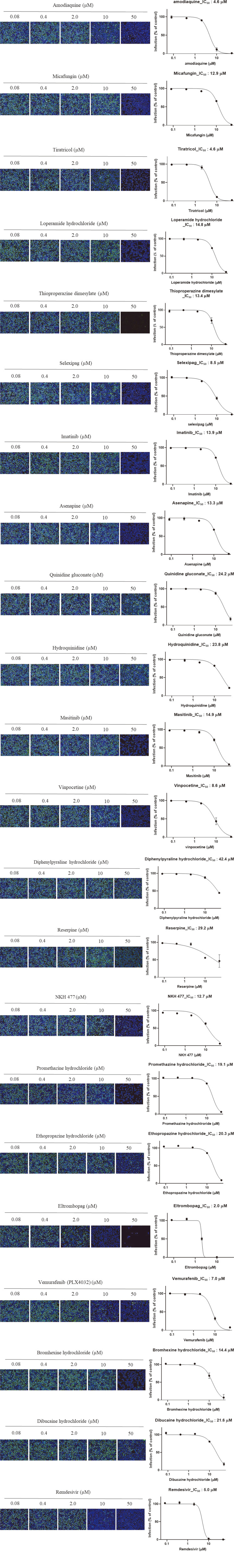
Dose-response curve of 21 compounds selected by the secondary screening with remdesivir as a positive control. The microscope images show cell nuclei (blue) and dsRNA (green) at each drug concentration.

To increase the cell survival differential between mock-infected cells and virus-infected cells, we used DMEM with low glucose and without fetal bovine serum as a dilution medium for both drug and virus. This method produced a Z’-factor of 0.62, which was considered adequate. Z’-factor is a statistical parameter that is used to assess the quality of the screening. After this optimization process, a secondary antiviral screen was performed. Twenty-six compounds showed at least 60% inhibition of virus-induced CPE. These were tested over a broader concentration range (0.08–50 μM). Among the 26 compounds, 21 showed antiviral activity based on immunofluorescent staining (Fig. 3). Their IC_50_ values are given in Table 2, with their cell toxicity (CC_50_) and selectivity index (SI) values. These 21 compounds were amodiaquine, micafungin, tiratricol, loperamide hydrochloride, thioproperazine dimesylate, selexipag, imatinib, asenapine, quinidine gluconate, hydroquinidine, masitinib, vinpocetine, diphenylpyraline hydrochloride, reserpine, NKH 477, promethazine hydrochloride, ethopropazine hydrochloride, eltrombopag, vemurafenib (PLX4032), bromhexine hydrochloride, and dibucaine hydrochloride.

Two compounds, amodiaquine and micafungin, were selected in both the primary and secondary screens. Amodiaquine is an anti-malaria drug, but it is also active against several viruses, including dengue virus, zika virus, MERS-CoV, and SARS-CoV [25-27]. Micafungin is an echinocandin with anti-fungal activity, which inhibits the β-1,3-D-glucan synthase of fungi [28, 29]. Antiviral activity against enterovirus and chikungunya virus has been reported [30, 31]. This is the first demonstration of antiviral activity of micafungin against SARS-CoV-2.

**Fig. 4:**
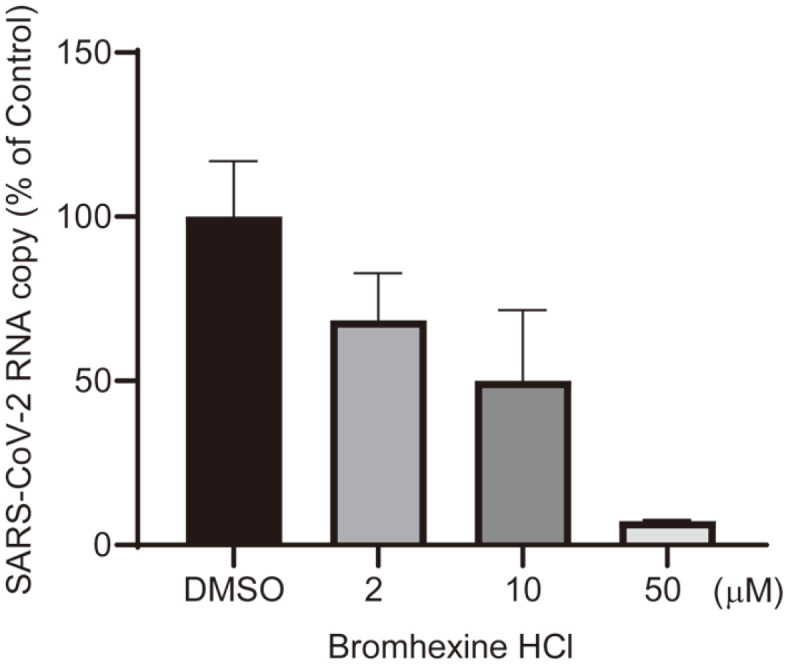
Suppression of SARS-CoV-2 replication by bromhexine hydrochloride. Cellular RNAs were isolated from cells infected by SARS-COV-2 and treated with bromhexine hydrochloride. The level of viral RNA was measured by real-time RT-PCR using primer sets specific to N gene of SARS-CoV-2.

Bromhexine hydrochloride is an inhibitor of TMPRSS2, a cofactor of SARS-CoV-2 entry, and one group reported that the drug was effective on COVID-19 patients in a clinical trial [32]. To further investigate whether bromhexine hydrochloride could inhibit SARS-CoV-2 replication, viral RNA was measured by real-time RT-PCR using primer sets specific to N gene of SARS-CoV-2. The treatment of this drug on virus-infected cells effectively reduced the intracellular RNA (Fig. 4).

Salinomycin and eltrombopag showed lower IC_50_ with good SI compared to remdesivir. Salinomycin, an antibiotic against gram-positive bacteria, also showed anti-viral effect on MERS-CoV and SARS-CoV [33, 34]. This drug was supposed to inhibit the endosomal acidification and the entry of viruses into cells. The thrombopoietin-receptor agonist eltrombopag has been used for the treatment of thrombocytopenia and showed antiviral effect on human cytomegalovirus via iron chelation [35]. There has been no report, however, on this drug having anti-viral activity against SARS-CoV or MERS-CoV. It would be interesting to investigate the target and mode-of-action of eltrombopag.

Overall, we identified 29 drugs that showed antiviral activity against SARS-CoV-2. Although further studies are required to understand their mode of action and confirm their efficacy in animal models, we now report this list of SARS-CoV-2 inhibitors to provide valuable basic information in the developmental support of potential therapeutic options.

## Figures and Tables

**Table 1:** Antiviral activity (IC_50_), cell toxicity (CC_50_), and selectivity index (SI) of 10 compounds selected by the primary screening with remdesivir as a positive control.

Drug name	IC_50_ (µM)	CC_50_ (µM)	Selectivity Index (SI)
Flunarizine	5.3	>100	>18.9
Micafungin	5.5	>100	>18.1
Amodiaquine	5.8	>100	>17.2
Meclizine	5.6	>100	>17.9
Lomerizine	12.8	>100	>7.8
Buclizine	19.6	>100	>5.1
Beclamide	72	>100	>1.4
Losartan	72	>100	>1.4
Salinomycin	0.58	56.3	97.1
Celecoxib	17.5	74.8	4.3
Remdesivir	5.0	>100	>20

**Table 2:** Antiviral activity (IC_50_), cell toxicity (CC_50_), and selectivity index (SI) of 21 compounds selected by the secondary screening with remdesivir as a positive control.

Drug name	IC_50_ (µM)	CC_50_ (µM)	Selectivity Index (SI)
Amodiaquine	4.6	>50	>10.9
Micafungin	12.9	>50	>3.9
Tiratricol	4.6	>50	>10.9
Loperamide hydrochloride	14.8	>50	>3.4
Thioproperazine dimesylate	13.4	41	3.1
Selexipag	8.5	>50	>5.9
Imatinib	13.9	>50	>3.6
Asenapine	13.3	>50	>3.8
Quinidine gluconate	24.2	>50	>2.1
hydroquinidine	23.8	>50	>2.1
Masitinib	14.9	47.1	3.2
Vinpocetine	8.6	>50	>5.8
Diphenylpyraline hydrochloride	42.4	>50	>1.2
Reserpine	29.2	>50	>1.7
NKH 477	12.7	>50	>3.9
Promethazine hydrochloride	19.1	>50	>2.6
Ethopropazine hydrochloride	20.3	>50	>2.5
Eltrombopag	2.0	43.8	21.9
Vemurafenib (PLX4032)	7.0	>50	>7.1
Bromhexine hydrochloride	14.4	>50	>3.5
Dibucaine hydrochloride	21.6	>50	>2.3
Remdesivir	5.0	>50	>10
